# Subitizing, unlike estimation, does not process sets in parallel

**DOI:** 10.1038/s41598-020-72860-4

**Published:** 2020-09-24

**Authors:** Wei Liu, Peng Zheng, Shaofang Huang, Guido Marco Cicchini

**Affiliations:** 1 College of Education, Dali University, Dali, China; 2 Institute of Neuroscience, National Research Council, Via Morruzi 1, 56124 Pisa, Italy; 3 College of Education, Yunnan Minzu University, Kunming, China

**Keywords:** Psychology, Human behaviour

## Abstract

Enumeration of very small quantities is a common task that we perform everyday. Much research has highlighted that in these conditions humans display fast, near errorless performance, a phenomenon dubbed subitizing. It has been suggested that this regime has a pivotal role in numerosity perception. Here we asked if this system can process multiple sets of items in parallel. At odds with what happens for moderate numerosities, we found a strong impairment caused already by the introduction of a second group of items marked by a different color. Adding shape as a cue provided no benefit. The only case in which subitizing was possible was when the target and distractor group were held constant through the experimental block. These results show the surprising fact that whilst being rapid and errorless, subitizing does not have the capability to disentangle multiple groups of items and deals only with coarse stimulus statistics.

## Introduction

Daily life presents uncountable occasions in which we need to estimate numerosity and even more so situations when we are faced with items of very low numerosity: the friends sitting with us at a restaurant table, the graduate students in the lab, or the red polos in a street market. Jevons^1^ was the first to observe that in these conditions (up to four items) we appraise rapidly and errorlessly the number of items, a phenomenon later dubbed subitizing by Kaufman and Lord^2^. Studies suggests that subitizing performance may be crucial to numerosity perception and acquisition of mathematical skills^3,4^ (but see the study of Anobile et al.^5^).

Despite much research has built on the notion that subitizing is a specific errorless regime, little is known about its basic features^6^. It is now quite clear that subitizing is distinct from the one regulating approximate estimation. Subitizing comes into play for sets of very few items (less than 5)^2^ and enables near errorless judgments (< 3%)^7^ which are performed rapidly regardless of the numerosity; whereas for higher numerosities estimation is performed only in an approximate manner and errors are proportional to numerosity (Weber’s law)^8,9^. At very low numerosities it is likely that both systems are active; typically the estimates of the subitizing system are preferred because they provide higher precision, however under attentional deprivation, the typical traits of the numerosity system, such as Weber’s law^10^ and susceptibility to previous stimuli emerge^11^.

One of the most salient traits of a perceptual systems is whether it can operate multiple estimates at the same time with little or no cost at all as this indicates an abundance of neural resources which operate in parallel^12,13^. This property has been tested for numerosities processed by the Approximate Number System (ANS) and it was found that even if two groups of dots are interspersed and subjects are told only in retrospective which group to enumerate, they pay very little cost for doing so^8^. This indicates that approximate numerosity proceeds in parallel, suggesting localized computational modules across the visual scene.

Similar evidence in the subitizing range, on the other hand, is limited to a couple of influential studies^7,14^. These authors have conducted a series of experiments in which subjects had to estimate targets among distractors. For example, targets were always white bars, and distractors were always black bars. With this paradigm they found that subjects performed rapidly and near errorlessly even in presence of distractors. However, given that the targets and distractors were constant throughout trials it is not clear whether subjects could optimize their selection of targets (and exclusion of distractors) on a session long basis. To address this point we replicated the paradigm of Halberda et al.^8^, in the subitizing regime. Subjects were presented with stimuli comprising multiple groups (from 1 to 3), defined by different colors, changing on every trial. In some trials subjects were instructed before stimulus presentation of the target color, and in other trials they were instructed after stimulus presentation. To anticipate the results we found that unlike approximate estimation, the ability to subitize is lost as soon as a second group is introduced in the scene. This is true even if subjects are informed in advance what will be the target group on every trial. Further control experiments revealed that subitizing occurs only if within a block the target group is kept constant, in the very condition used by Trick and collaborators^7,14^.

Overall these results point to a strong limitation of the mechanism which enumerates few items errorlessly and re-frame it as an extremely specific process which produces errorless performance only with simple sets, that require no segregation.

## Method

### Sample size and subjects

The main dependent variable is CV, the ratio between a standard deviation (SD) of estimation and the target number and returns a dimensionless measure of noise of the judgments. This is a dimensionless quantity that allows comparison of performance across numerosities. Given that our observers performed unbiased estimates we have preferred it to Weber fraction (i.e. the ratio between measured standard deviation and measured average estimates) as its measurement error depends only on estimation error of SD leaving out that associated with the average estimate.

CV is not a standard measure included in software packages. To calculate the number of trials needed for statistics we ran simulations anticipating that two distinct statistical comparisons could be involved. In one scenario we asked the minimum number of trials and subjects necessary to report statistical significance (i.e. *Q* < 0.05) between two conditions with CVs between 0.2 and 0.35 in 90% of the cases, a situation similar to the one found by previous study^8^ when comparing “probe-before” and “probe-after” in the three-color conditions. In a second comparison we asked how many subjects and trials were necessary to document a difference in CVs between 0.05 and 0.2 (assuming one had to compare a subitizing performance and a typical estimation performance) with a power of 0.90.

In our simulations we checked various combinations of number of trials and observers. For each simulation we run 10,000 virtual experiments with the observers running at the two noise levels and checked if statistical significance was reached in each iteration. As the task implies quantized estimates, the measured variance depends also to how close the mean estimate of observer is to the boundary between the two integers. In order to incorporate all possible cases we allowed each observer to have a bias spanning all possible values (i.e. from − 0.5 to + 0.5 from the correct target value).

A sufficient power was obtained with several combinations of number of trials and subjects. One that struck the balance between suggested to show 9 trials per condition to 10 observers in the first comparison (CV 0.2 versus CV 0.35) and 5 trials per condition in the second scenario (comparison of 0.05 and 0.2). Such figures may look quite unusual for psychophysical experiments. However it is worth considering that an unbiased observer performing only 5 estimates in the subitizing regime (with a CV of 0.05) returns a streak of identical estimates (with zero SD) 99.7% of the times, a rather distinguishable trait. In contrast, an observer has an uncertainty of 20% (CV of 0.20), will yield non-zero SD in 93% of the cases. This effect varies to a degree if the observer is biased or not; however, it exerts a strong drive onto statistical comparisons both at individual and at a group level.

Subjects aged from 19 to 36 years with normal or corrected-to-normal vision and normal color perception were recruited. 13 subjects (averagely aged 28.9 years, 6 males) participated in Experiment 1. A total of 3960 trials were run, and 3172 trials in which the target number was in the range of 6–28 were analyzed in Experiment 1A. A total of 4966 trials were run, and 2753 trials within target number range 1–4 were analyzed in Experiment 1B. A similar number of trials were run in Experiment 1C. 12 subjects (averagely aged 25 years, 5 males) participated in Experiment 2. A total of 12,096 trials were run, and 6910 trials were analyzed. Sufficient trials were provided in each condition.

### Stimuli and procedure

Subjects sat approximately 50 cm from an LCD monitor with a viewable area measuring 41 cm by 26 cm (19″, 1920 × 1080, 60 Hz) in a dimly lit quiet room. Stimuli were generated from Matlab (Mathworks, Natwick, MA). In Experiment 1, the diameter of each dot is 0.5° visual angle (20 pixel). Dots and masks were presented in a circle at the center of the screen with a diameter of 15° (600 pixel) visual angle.

The paradigm of Experiment 1 followed that of previous study^8^. On each trial, subjects saw a 500-ms display containing 18–29 dots of one to five colors (red, green, blue, yellow, and magenta). They enumerated the number of the target, which could be one of the color subsets or the superset. At the beginning of each trial, a fixation was shown for 500 ms. Then, the probe screen which indicated the target was presented, followed by stimulus screen and another probe screen. Each presentation lasted for 500 ms. The stimulus was straddled by two 500-ms masks, one preceding and the other following it. Masks always comprised 300 dots in all the 5 colors and spanned the whole circular region. Subjects typed their answers (any number they wished) into computer after the onset of the second probe.

**Figure 1:**
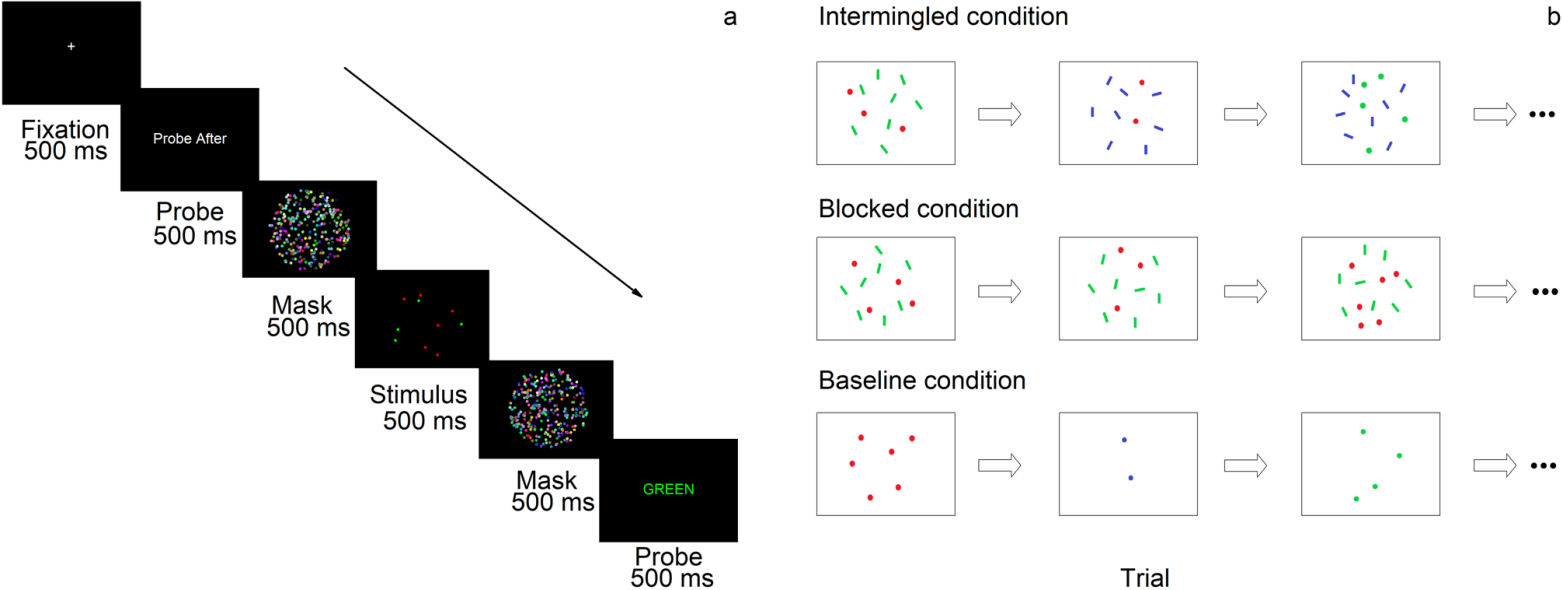
(**a**) Schematic illustration describing the procedure of Experiment 1B. A fixation lasted for 500 ms at the beginning of each trial. Then, probe screen was presented, followed by stimulus screen and another probe screen, each lasting for 500 ms. These screens were straddled by two mask screens. On probe-before trials, the target was presented in the first “Probe” screen, whereas on probe-after trials, the target was probed in the second one. Subjects typed their answers into computer after the onset of the second probe. (**b**) Illustration of the conditions of Experiment 2. Subjects should always report the number of dots. In the intermingled condition, stimulus colors were changed on every trial, whereas they were constant in the blocked condition. The baseline condition comprised no bars. White background and absence of masks were adopted to replicate the paradigm of previous study^7^.

In each of the subset/superset group, there were two conditions. In probe-before condition, the target was presented in the first “Probe” screen. In probe-after condition, the first screen just stated that the target would be revealed later and the target was specified in the second “Probe” screen, after the presentation of the stimulus (Fig. 1a). Within each subset/superset group, the proportion and target number range of these two conditions are approximately equal.

Dots in stimulus patch were randomly distributed with the constraint that they could not overlap with each other. The number of dots in each color subset was randomly determined, and in half (49.6%) of the trials the target subset was smaller than at least one distracting subset, making the strategy of attending only to the largest subset ineffective. For each subject, an average of 315 trials which combined all conditions in a randomized order were run in 4 blocks in Experiment 1A. Generally, on 22% of the trials, subjects were asked to report the overall number of the dots regardless of the colors (superset trials). The proportion of one-, two-, and three-color subset conditions was 19%, 32%, and 21%, respectively. In 2% of the trials, there were four or five color subsets. Proportion of each condition is slightly different among Experiment 1A, B, and C.

Experiment 1B was similar to 1A except that the overall dot number is 1–12. Occasionally, in 4% of the trials, there was no target color represented, and the correct respond is ‘0’. These trials were designed to prevent subjects from making less mistakes to target ‘1’.

Experiment 1C was conducted to investigate whether additional feature such as shape can help observers in grouping and subitizing. Each dot subset possessed both an identical color and an identical shape. Dots were twice bigger in size than those in Experiment 1B to underline the feature of shape.

In Experiment 2, On each trial, subjects saw a 250-ms display containing 1–7 dots of identical color with distracting bars with a fixed number (6–10 bars were presented respectively in each block) in other color (Fig. 1b). The diameter of each dot is 0.3° visual angle (12 pixel), and the size of each bar is 6 × 18 pixel. The occupied area is 108 pixel both for each dot and each bar. Dots and bars were randomly distributed against white background in a circle at the center of the screen with a diameter of 15° (600 pixel) visual angle. Dots would never be overlapped by bars or other dots. In two-group intermingled condition, the colors of dots and bars were randomly decided (red, green, or blue) on each trial. Subjects were aware that dots and bars always had different colors, and they should enumerate dots, which could be separated from bars both by color and shape features. At the beginning of each trial, a fixation was shown for 500 ms, followed by a blank screen for 500 ms. Then, the stimuli were presented for 250 ms, followed by a blank screen lasting for 1750 ms. Subjects reported their answers verbally. Their answers were recorded and typed into computer after the experiment. In two-group blocked condition, dots were always red, and bars were always green. Subjects were aware of that before they began. No bars were presented in baseline condition.

### Data analysis

CV, mean, and error rate of estimation were calculated. Linear regression was conducted with mean of estimation as independent variable and standard deviation (SD) as dependent variable. Two variants of models were compared, one complying with simple Weber’s law (a linear fit without intercept) and the other complying with modified Weber’s law (a linear fit with positive intercept)^15^, performing the *F* test. In this study, one-sample *t* tests were one-tailed, and paired *t *tests were two tailed. The False Discovery Rate probability (FDR), or *Q* value, is adopted in this study to correct probability of type-I error in multiple comparisons^16^. *Q* = 0.05 is a widely accepted threshold for significance. For more details refer to Supplementary online.

### Statement of ethical approval

For both of the experiments, the data were analyzed anonymously. All participants provided their informed consent according to the Declaration of Helsinki prior to the experiment in both verbal and written forms, and they were compensated for their participation. Yunnan Minzu University’ s ethics committee approved this study.

## Results

### Experiment 1

**Figure 2:**
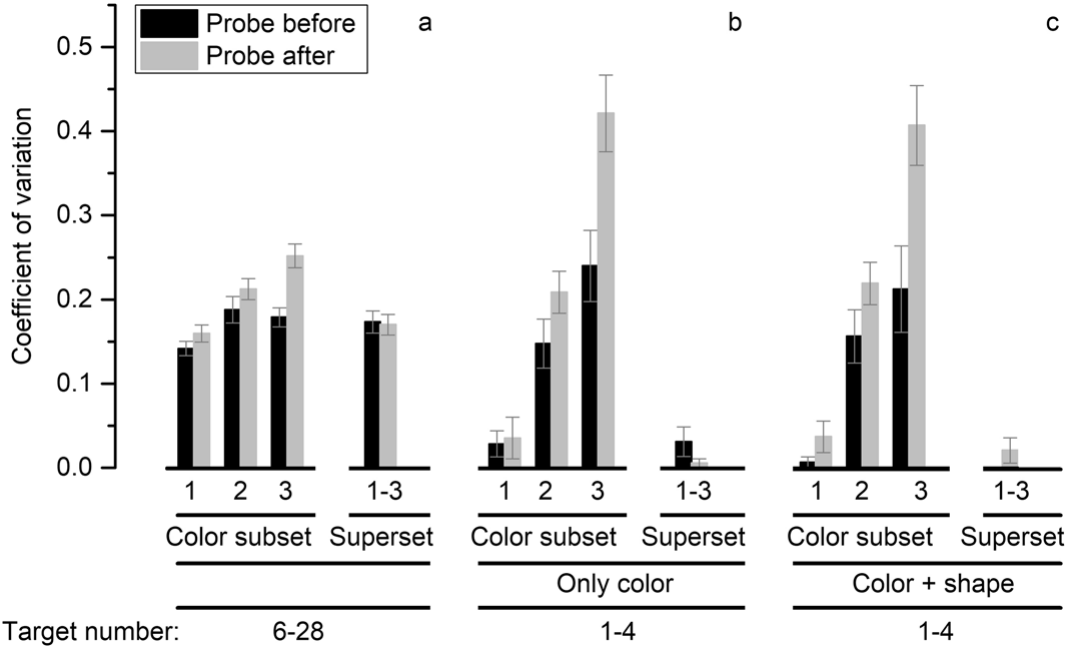
Results in Experiment 1. The bar graph presents average coefficient of variation (CV) for probe-before (black) and probe-after (gray) trials. For color subset trials, on which only a single subset was enumerated, CV is graphed against the number of colors in the array; for superset trials, on which subjects enumerated all the dots in the display, CV of 1–3 color(s) is plotted together. Error bars show standard errors. For significance refer to text. (**a**) The results within estimation range. (**b**) The results within subitizing range with color clue. (**c**) The results within subitizing range with conjunction clues (color and shape).

Figure 2 displays CVs in different conditions. As for the estimation range (Fig. 2a), One-way repeated measures ANOVA with ‘before’ conditions as independent variable shows that there is no significant difference among probe-before conditions, *F*(3, 36) = 2.65, *p* = 0.063, *η*^2^ = 0.14, *BF*_10_ = 1.72. Subjects can estimate dots either in the color subset or in superset as efficient as they estimate dots in single-color set.

The differences of CVs between probe before and after conditions are not significant in two-color subset group, *t*(12) = 2.15, *Q* = 0.083, *d* = 0.60, *BF*_10_ = 1.56, and in superset group, *t*(12) = 0.21, *Q* = 0.835, *d* = 0.06, *BF*_10_ = 0.28. Significant difference occurs in three-color group, *t*(12) = 3.79, *Q* = 0.003, *d* = 1.05, *BF*_10_ = 17.89, indicating that subjects could not enumerate effectively the number of items of the target color when the target is told after the presentation of stimulus, as opposed to the probe-before condition. These results replicate previous study^8^ in particular subjects display no cost when they were told after stimulus presentation whether they had to enumerate one of two subsets or even the superset. This indicates that they can perform three numerosity estimations simultaneously and spontaneously. For more statistics refer to Supplementary online (Table S1; S2).

As to the range of 1–4 (Fig. 2b), CVs in one-color conditions are below 0.04 and are not significantly larger than ‘0’ both on probe before and after trials, and error rates are below 3% (Supplementary, Table S1). These results are consistent with the idea that subitizing is engaged and performance is essentially error-free.

In the multiple-color subset group, however CVs raise considerably as soon as a second group is introduced and range between 0.15 to 0.43 across conditions. Error rates range from 8.1% to 36.7% in these conditions. (Supplementary, Table S1). It is clear that errorless subitizing is absent. When there are multiple color subsets in the visual field, subjects cannot subitize them without a tangible cost, even when they are told in advance which one is the target.

CVs are not significantly different between probe-before and after trials in two-color conditions, whereas it is significantly different between three-color probe-before and after conditions (Supplementary, Table S2). This performance pattern (i.e. CVs of about 0.20 in two-color and three-before conditions and a further decrement of performance in the three-after condition) is strongly reminiscent of the data pattern within the moderate numerosity range of Experiment 1A.

In superset condition, again, CVs are not significantly larger than ‘0’, both on probe before and after trials (Supplementary, Table S1). CVs in superset are significantly different from those of multiple-color groups, whereas they are not different from those in one-color groups (Supplementary, Table S1; S2). Thus, subjects can subitize if they are asked to enumerate all dots, irrespective of probe conditions and the colors defining each group.

To check if the results in multiple-color conditions reflect a genuine result of the coming into play of the estimation system, which obeys Weber’s law, we plotted SD of estimation against the mean estimation for each target number and performed linear regression (see Supplementary, Figure S1). As expected within the range of 6–28, linear regression analysis reveals significant effects of numerosity on SD across all conditions. The slopes are all significantly different from ‘0’ (*p* < 0.001) with values varying from 0.15 to 0.31. This confirms that Weber’s law is valid when estimation mechanisms are active within the range of 6–28.

**Figure 3:**
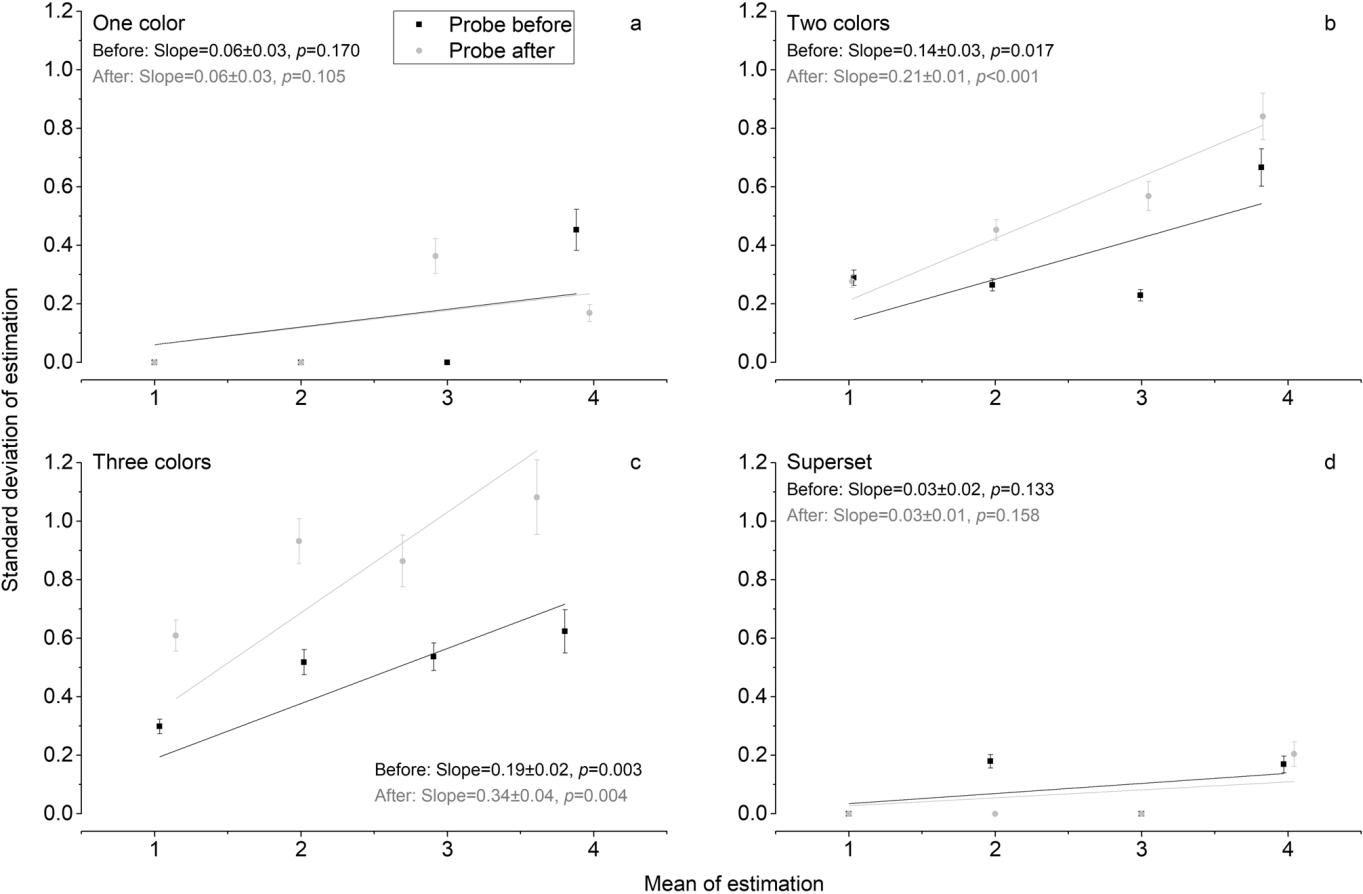
Scatter and linear regression results in Experiment 1B. The line graphs present linear regression results of estimation for probe-before (black lines) and probe-after (gray lines) trials. Standard deviation (SD) is plotted against mean of estimation within number range 1–4. Error bars show standard errors. Slope values were shown with standard errors in each condition.

As to the subitizing range, slopes in one-color subset and superset conditions are not significantly different from ‘0’ (Fig. 3). Crucially, in the multiple-color conditions which depart from subitizing, errors also depend on numerosity hinting at the presence of Weber’s law. Slopes in these conditions are all significantly different from ‘0’ (*p* < 0.020), ranging from 0.14 to 0.34, both on probe-before and after trials.

In Experiment 1C, we paired every subset with a given color and a specific shape to investigate whether additional feature such as shape can help observers in subitizing. However, the same pattern of results was obtained (Fig. 2c). CVs in one-color subset and superset (CV < 0.04) are not significantly different from ‘0’, revealing subitizing, whereas they vary from 0.16 to 0.41 and the slopes are significantly different from ‘0’ in multiple-color conditions (see Supplementary, Table S1; S1; and Figure S2), confirming Weber’s law.

### Experiment 2

Results in Experiment 1 (B; C) seem at odd with the classic finding^7^ which suggests that subitizing is possible in presence of distracting elements. One key feature however of that study is that the target group was held constant across an experimental block, whereas our paradigm prescribes a new target on every trial. To investigate whether constant target is crucial in triggering subitizing, we ran Experiment 2 with at most two color subsets and targets that could be either changed on every trial or blocked across the session (Fig. 1b).

CV in one-color baseline condition is not significantly different from 0, *t*(11) = 1.00, *p* = 0.339, *d* = 0.29, *BF*_10_ = 0.44, indicating a hallmark of subitizing. In the target intermingled condition, however CV raises, *M* = 0.11, *SD* = 0.04, 95% confidence interval or CI = [0.08, 0.13], which is significantly different from that of baseline, *t*(11) = 8.21, *p* < 0.001, *d* = 2.37, *BF*_10_ > 100. In the target blocked condition, instead, CV is again very low, *M* = 0.03, *SD* = 0.02, 95% CI = [0.02, 0.04], which is significantly different from the intermingled condition, *t*(11) = 9.43, *p* < 0.001, *d* = 2.72, *BF*_10_ > 100, as well as from baseline, *t*(11) = 3.47, *p* = 0.005, *d* = 1.00, *BF*_10_ = 10.04. All these results occur on the face of no bias in the estimates (see Supplementary).

To exclude the possibility that other factors such as salience or contrast could account for the CV difference between the intermingled and blocked conditions, we analyzed the trials in the intermingled condition with the very same colors employed in the blocked condition. Also on these trials, CV is significantly different from that in the blocked condition, *M* = 0.09, *SD* = 0.06, 95% CI = [0.05, 0.13]; *t*(11) = 4.12, *p* = 0.002, *d* = 1.19, *BF*_10_ = 25.59.

**Figure 4:**
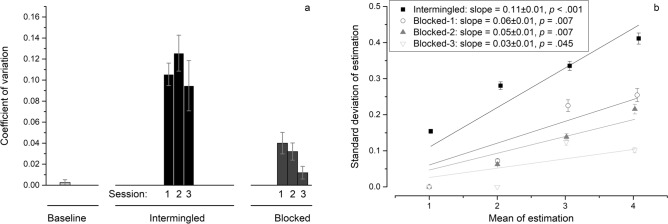
Results in Experiment 2. (**a**) The bar graph presents average CV of each session in each condition. Session 1, 2, and 3 stand for the first 64, middle 106, and last 64 trials for each subject in each condition. Error bars show standard errors. For significance refer to text. (**b**) The line graphs present linear regression results of estimation for the intermingled (rectangle) and blocked (circle, triangle, and inverted triangle stand for Session 1–3 respectively) conditions. SD is plotted against mean of estimation. Error bars show standard errors.

To figure out whether subitizing-like pattern can be achieved by practice, we analyzed the CV and slope values in different period (Fig. 4). Session 1, 2, 3 stands for the first 64, middle 106, and last 64 trials for each subject. In the intermingled condition there is no significant difference among three sessions. In the blocked condition, however significant differences exist between the last session and the former sessions (see Supplementary). CV in Session 3, *M* = 0.01, *SD* = 0.02, 95% CI = [0.00, 0.03], is not significantly different from baseline, *t*(11) = 1.54, *p* = 0.151, *d* = 0.45, *BF*_10_ = 0.74, indicating that when targets are consistent across trials, subjects gradually manage to subitize as they repeat the tasks. A decrease in slope is also revealed in linear regression.

## Discussion

Halberda et al.^8^ pointed out that as to most subjects, visual attention is efficient for selecting and enumerating the number of three sets in parallel: two color subsets and the superset, which is compatible with the three-item limits of object-based attention^17^. This evidence was collected with a number range of 7–30 and a brief presentation time. Our work replicated these results in Experiment 1A and attempted extending these findings to numerosities of very few items which trigger a specific regime of subitizing. Within the range of 1–4, it was found that subitizing only exists in the one-color and superset conditions, whereas it is absent in multiple-color conditions. Including of additional grouping information, such as shape, will not help engaging subitizing.

This is not due to a generic limit of working memory^18^ as, with higher numerosities, up to three qualities can be estimated without cost. Similarly, this result cannot be due to the inability to direct attention towards multiple groups as this is possible for moderate numerosities. Neither the results can be ascribed to a low level degradation of inputs caused by the distractors as the blocked condition of experiment 2 yields near errorless performance. We rather suggest that this reflects a specific limitation of the subitizing system.

It is now firmly accepted that the availability of attentional and cognitive resources is a precondition for subitizing^10,19,20^. At the same time the availability of attentional resources has a limited impact on approximate estimation^20–22^. This makes it an ideal candidate to explain why subitizing cannot operate on multiple sets whilst approximate estimation can; it suffices to assume that the simple request to segregate the visual scene into two groups by itself drains resources critical for subitizing. This hypothesis however, cannot explain why in the probe-after condition subjects can subitize the superset. In this condition subjects are told after stimulus (and mask) presentation which items they have to enumerate and likely they perform some degree of segregation between groups as this is necessary for the two and three subset trials. Yet subjects perform near flawlessly. The only possibility to reconcile this data within the attentional demand framework is to assume that the superset has some form of invulnerability to the availability of attentional resources. This is something that has been postulated before for gist perception^23^ (but see the study of Cohen et al.^24^). Only future experiments may test if this is true for numerical judgments of superset.

Interestingly the introduction of multiple groups impacts subitizing on the subgroups but not on enumerating the whole set. The errorless performance of enumerating “all” cannot be attributed to the result of enumerating the subsets which contains one or two dots respectively and summing them up, either. Further analysis showed that even when the total number of dots in the visual field was no more than four in Experiment 1B, subjects still made mistakes in enumerating subsets: CV = 0.12 on probe-before trials; CV = 0.18 on probe-after trials which clearly would predict high CVs in the superset condition. These results indicate that there is a precise perceptual difference between subset and superset, and that the superset is processed by an efficient perceptual subsystem. Interestingly the advantage of enumerating “all” was also revealed in estimation: on probe-after trials, even when potential target sets number was three and beyond, the estimation of the superset did not drop on probe-after trials (the current study; the study of Halberda et al.^8^).

In Experiment 2, subjects were asked to enumerate dots, and they were aware that target and distractors were distinguishable both in color and shape. When colors are changed from trial to trial in the intermingled condition subitizing is lost in favour of approximate estimation with a CV of 0.11. When the targets are blocked and target color are consistent across trials, enumeration improves significantly. Notably the error rate in our study and previous study^7^ are very similar (1.6% in ours, 1.9% in theirs) indicating that, despite some difference in paradigm (presentation time, choice of color and shape) our setup tapped on the same mechanism. As trial number increases in the blocked condition, subjects gradually show an error-free pattern in their enumeration, underlining also a role of practice in this condition. The overall conclusion is that previous findings which documented that subitizing could occur also in complex displays, were in fact collected in rather favorable conditions.

Our research also reveals that when subitizing cannot be performed, estimation is carried out with a reasonable precision. In these conditions, two crucial markers suggest that the process of estimation is taken over by the mechanisms that subtend the ANS. The first is that the CVs are close to 0.14–0.20 which is the typical resolution of the ANS^6^. The second is that errors comply to Weber’s law^8,9,25^. Thus, similarly to what is found for attention deprivation^10^, we found that the ANS can also operate on very low numerosities and is ready to be engaged whenever the subitizing cannot function properly.

Overall, our research shows that subitizing is rather fragile, especially if compared with the mechanisms of approximate estimation. The mere introduction of a second color is sufficient to proscribe subitizing even when subjects knew which target they had to concentrate on before the onset of stimulus. This indicates that whilst errorless subitizing parses only coarse stimulus statistics and has no access to the identity of subgroups of items in spatial overlap.

## Supplementary information

Supplementary Information.

## Data Availability

The data sets generated and analyzed during the current study are available from the corresponding author on reasonable request.
