# Supplementary material for: Subitizing, unlike estimation, does not process sets in parallel
Source: Sci Rep. 2020 Sep 24;10:15689. doi: 10.1038/s41598-020-72860-4 (PMC7518424; doi:10.1038/s41598-020-72860-4)
Supplement: Supplementary file 1 — Supplementary Information. [file 41598_2020_72860_MOESM1_ESM.doc]

**Subitizing, unlike estimation, does not process sets in parallel**

**Wei Liu^1, 3^, Peng Zheng^3^, Shaofang Huang^3^, and Guido Marco Cicchini^2^***

1 College of Education, Dali University, Dali, China

2 Institute of Neuroscience, National Research Council, Pisa, Italy

3 College of Education, Yunnan Minzu University, Kunming, China

*Corresponding Author: **Guido Marco Cicchini**

Tel.: +393771243180

Email: cicchini@in.cnr.it

Address: Institute of Neuroscience, National Research Council, Via Morruzi 1, 56124, Pisa, Italy

1. **Data analysis**

JASP version 0.9.2 and SPSS version 24 were used for statistics. Two linear regression models, one with intercept and the other without, were compared performing the *F*-test in OriginPro 2015 (www.originlab.com). In all conditions the model which yields a more parsimonious fit of the data was the one without intercept. On nine trials throughout the current study, original responses were extreme (larger than—and implies that subjects mistook the distractors for targets). These responses were modified to 0 in line with the fact that subjects missed the targets. No other data was excluded.

1. **Results**
   1. **Experiment 1**

**Table S1.** Descriptive statistics of Experiment 1.

| Experiment | Statistics | One Color | | Two Colors | | Three Colors | | Superset | |
| --- | --- | --- | --- | --- | --- | --- | --- | --- | --- |
|  |  | Before | After | Before | After | Before | After | Before | After |
| **1A** | *M* | 0.14 | 0.16 | 0.19 | 0.21 | 0.18 | 0.25 | 0.17 | 0.17 |
|  | *SD* | 0.03 | 0.04 | 0.06 | 0.04 | 0.04 | 0.05 | 0.05 | 0.04 |
|  | 95% CI | [0.12, 0.16] | [0.14, 0.18] | [0.15, 0.22] | [0.19, 0.24] | [015, 0.20] | [0.22, 0.28] | [0.14, 0.20] | [0.14, 0.20] |
| **1B** | *M* | 0.03 | 0.04 | 0.15 | 0.21 | 0.24 | 0.42 | 0.03 | 0.01 |
|  | *SD* | 0.06 | 0.09 | 0.11 | 0.09 | 0.15 | 0.16 | 0.06 | 0.02 |
|  | 95% CI | [-0.01, 0.06] | [-0.02, 0.09] | [0.08, 0.21] | [0.15, 0.26] | [0.15, 0.33] | [0.32, 0.52] | [-0.01, 0.07] | [-0.01, 0.02] |
|  | *p*(CV>0) | .046 | .090 | <.001 | <.001 | <.001 | <.001 | .052 | .169 |
|  | *Q*(CV>0) | .074 | .103 | <.001*** | <.001*** | <.001*** | <.001*** | .069 | .169 |
|  | *BF_10_*(CV>0) | 1.93 | 1.13 | >100 | >100 | >100 | >100 | 1.74 | 0.69 |
|  | Error Rate | 2.1% | 2.1% | 8.1% | 18.1% | 13.7% | 36.7% | 2.9% | 0% |
| **1C** | *M* | 0.01 | 0.04 | 0.16 | 0.22 | 0.21 | 0.41 | 0 | 0.02 |
|  | *SD* | 0.02 | 0.07 | 0.11 | 0.09 | 0.18 | 0.17 | 0 | 0.05 |
|  | 95% CI | [-0.01, 0.02] | [0.00, 0.08] | [0.09, 0.23] | [0.16, 0.27] | [0.10, 0.32] | [0.30, 0.51] | [0, 0] | [-0.01, 0.05] |
|  | *p*(CV>0) | .169 | .036 | <.001 | <.001 | .001 | <.001 | - | .096 |
|  | *Q*(CV>0) | .193 | .058 | <.001*** | <.001*** | .001** | <.001*** | - | .128 |
|  | *BF_10_*(CV>0) | 0.69 | 2.34 | >100 | >100 | 61.60 | >100 | - | 1.08 |
|  | Error Rate | 0.5% | 2.3% | 7.8% | 17.5% | 13.5% | 43.4% | 0% | 1.4% |

Note: *M*, *SD*, and 95% CI stand for mean, standard deviation, and 95% confidence interval of coefficient of variation (CV). *Q* value stands for the False Discovery Rate probability (FDR), or the corrected probability of type-Ⅰerror in multiple comparisons. **Q*<.05, ***Q*<.01, ****Q*<.001.

In Experiment 1A, CVs are not significantly different among probe-before conditions, *F*(3, 36) = 2.65, *p* = .063, *η^2^* = 0.14, BF_10_ = 1.72. A significant difference is revealed among probe-after conditions, *F*(3, 36) = 13.22, *p* < .001, *η^2^* = 0.42, BF_10_ > 100. In Experiment 1B, there is a significant difference among probe-before conditions, *F*(3, 36) = 12.05, *p* < .001, *η^2^* = 0.45, BF_10_ > 100, and among probe-after conditions, *F*(3, 36) = 41.98, *p* < .001, *η^2^* = 0.73, BF_10_ > 100. In Experiment 1C, there is a significant difference among probe-before conditions, *F*(3, 36) = 14.55, *p <* .001, *η^2^* = 0.44, BF_10_ >100, and among probe-after conditions, *F*(3, 36) = 53.66, *p* < .001, *η^2^* = 0.70, BF_10_ > 100.

Results of paired-*t* tests between each two conditions are shown in Table S2. In probe-after condition of Experiment 1A, there is a significant difference between one- and two-color groups, and no significant difference between two- and three-color groups. It is still possible that some of the subjects manage to enumerate three sets in parallel, whereas others can only enumerate two (one subset and superset).

**Table S2.** Multiple comparison results of Experiment 1.

| **Experiment 1A** | **One-color** | **Two-color** | **Three-color** | **Superset** |
| --- | --- | --- | --- | --- |
| **One-color** | *t*(12)=1.33, p=.209, d=0.37, BF_10_=0.57  *Q*=.304 | *t*(12)=2.40, p=.034, d=0.67, BF_10_=2.20  *Q*=.078 | *t*(12)=2.86, p=.014, d=0.79, BF_10_=4.32  *Q*=.045* | *t*(12)=2.69, p=.020, d=0.75, BF_10_=3.37  *Q*=.053 |
| **Two-color** | *t*(12)=3.99, p=.002, d=1.11, BF_10_=24.26  *Q*=.002** | *t*(12)=2.15, p=.052, d=0.60, BF_10_=1.56  *Q*=.083 | *t*(12)=0.44, p=.667, d=0.12, BF_10_=0.30  *Q*=.762 | *t*(12)=0.69, p=.501, d=0.19, BF_10_=0.34  *Q*=.617 |
| **Three-color** | *t*(12)=5.47, p<.001, d=1.52, BF_10_=210.64  *Q*<.001*** | *t*(12)=2.26, p=.043, d=0.63, BF_10_=1.81  *Q*=.078 | *t*(12)=3.79, p=.003, d=1.05, BF_10_=17.89  *Q*=.003** | *t*(12)=0.32, p=.757, d=0.09, BF_10_=0.29  *Q*=.807 |
| **Superset** | *t*(12)=0.81, p=.434, d=0.23, BF_10_=0.37  *Q*=.579 | *t*(12)=2.33, p=.038, d=0.64, BF_10_=1.99  *Q*=.078 | *t*(12)=4.31, p=.001, d=1.19, BF_10_=38.86  *Q*=.001** | *t*(12)=0.21, p=.835, d=0.06, BF_10_=0.28  *Q*=.835 |
| **Experiment 1B** | **One-color** | **Two-color** | **Three-color** | **Superset** |
| **One-color** | *t*(12)=0.23 p=.824, d=0.06, BF_10_=0.29  *Q*=.879 | *t*(12)=3.91, p=.002, d=1.08, BF_10_=21.30  *Q*=.002** | *t*(12)=4.49, p<.001, d=1.25, BF_10_=51.45  *Q*<.001*** | *t*(12)=0.11, p=.914, d=0.03, BF_10_=0.28  *Q*=.914 |
| **Two-color** | *t*(12)=4.18, p=.001, d=1.16, BF_10_=31.97  *Q*=.001** | *t*(12)=1.56, p=.145, d=0.43, BF_10_=0.74  *Q*=.193 | *t*(12)=1.63, p=.129, d=0.45, BF_10_=0.80  *Q*=.188 | *t*(12)=4.61, p<.001, d=1.28, BF_10_=60.87  *Q*<.001*** |
| **Three-color** | *t*(12)=7.17, p<.001, d=1.99, BF_10_>1000  *Q*<.001*** | *t*(12)=4.40, p<.001, d=1.22, BF_10_=45.04  *Q*<.001*** | *t*(12)=3.13, p=.009, d=0.87, BF_10_=6.46  *Q*=.014* | *t*(12)=3.95, p=.002, d=1.10, BF_10_=22.81  *Q*=.002** |
| **Superset** | *t*(12)=1.20, p=.252, d=0.33, BF_10_=0.51  *Q*=.288 | *t*(12)=7.51, p<.001, d=2.08, BF_10_>1000  *Q*<.001*** | *t*(12)=8.94, p<.001, d=2.48, BF_10_>1000  *Q*<.001*** | *t*(12)=1.34, p=.206, d=0.37, BF_10_=0.58  *Q*=.254 |
| **Experiment 1C** | **One-color** | **Two-color** | **Three-color** | **Superset** |
| **One-color** | *t*(12)=1.94, p=.076, d=0.54, BF_10_=1.17  *Q*=.101 | *t*(12)=4.43, p<.001, d=1.23, BF_10_=46.96  *Q*<.001*** | *t*(12)=3.99, p=.002, d=1.11, BF_10_=24.28  *Q*=.002** | *t*(12)=1.00, p=0.337, d=0.28, BF_10_=0.42  *Q*=.359 |
| **Two-color** | *t*(12)=7.78, p<.001, d=2.16, BF_10_>1000  *Q*<.001*** | *t*(12)=2.13, p=.055, d=0.59, BF_10_=1.50  *Q*=.080 | *t*(12)=1.26, p=.232, d=0.35, BF_10_=0.54  *Q*=.265 | *t*(12)=4.92, p<.001, d=1.37, BF_10_=96.50  *Q<*.001*** |
| **Three-color** | *t*(12)=7.82, p<.001, d=2.17, BF_10_>1000  *Q*<.001*** | *t*(12)=4.57, p<.001, d=1.27, BF_10_=57.86  *Q*<.001*** | *t*(12)=3.54, p=.004, d=0.98, BF_10_=12.10  *Q*=.006** | *t*(12)=4.15, p=.001, d=1.15, BF_10_=30.84  *Q*=.001** |
| **Superset** | *t*(12)=0.10, p=.339, d=0.28, BF_10_=0.42  *Q*=.359 | *t*(12)=8.57, p<.001, d=2.38, BF_10_>1000  *Q*<.001*** | *t*(12)=8.49, p<.001, d=2.35, BF_10_>1000  *Q*<.001*** | *t*(12)=1.38, p=.192, d=0.38, BF_10_=0.61  *Q*=.236 |

Note: In each experiment, horizontal and vertical columns stand for the two conditions been compared. The triangle areas with white/dark gray background demonstrate the comparisons between two conditions on probe-before/after trials.


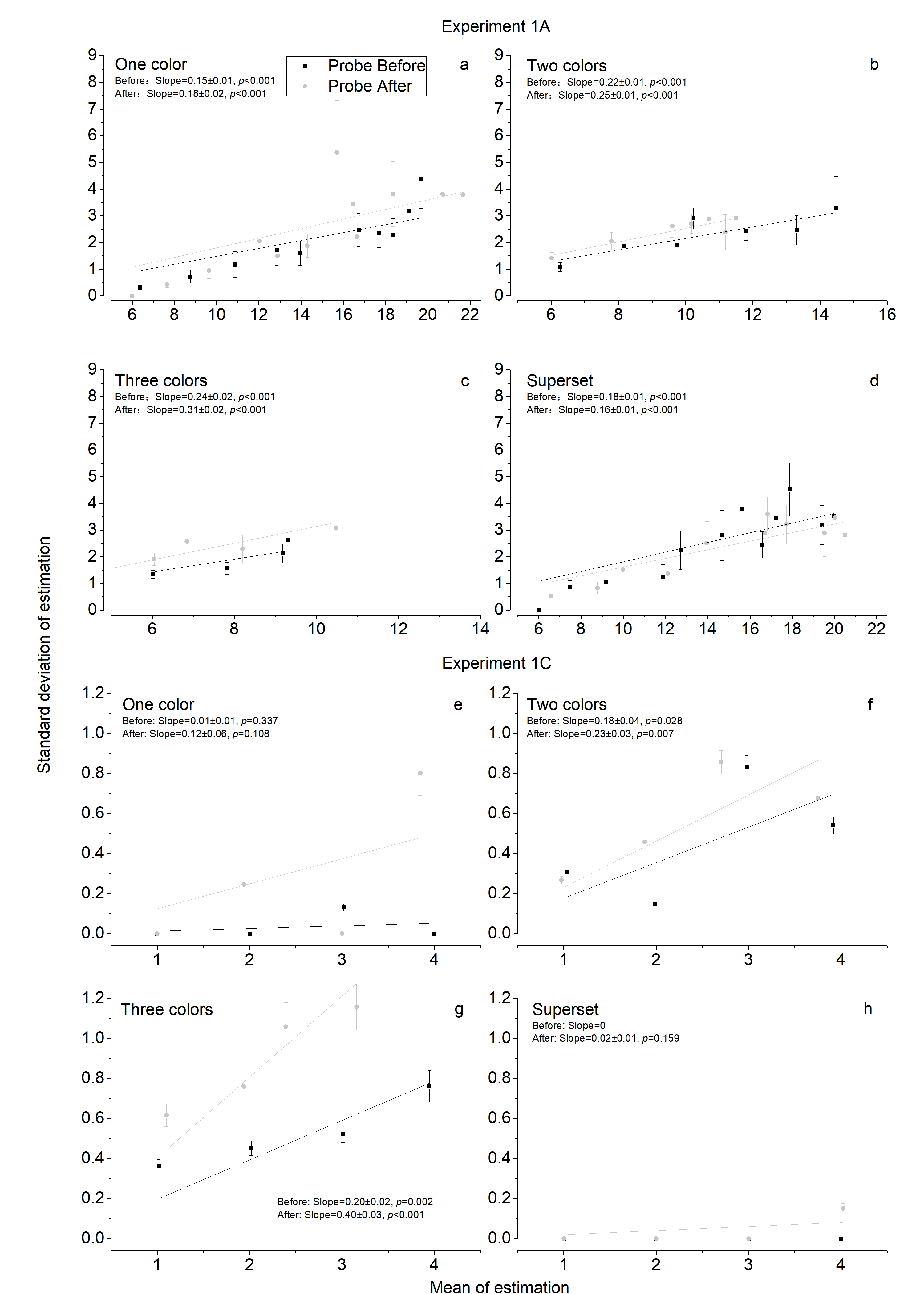


**Fig. S1.** Scatter and linear regression results in Experiment 1A (a-d) and 1C (e-h). Standard deviation (SD) is plotted against mean of estimation. Data from the probe-before/after conditions are displayed in black/gray. Error bars show standard errors. The line graphs indicate linear regression. (a-d) The target number which appeared on only one or two trials were excluded to eliminate unreliable SD values. Number range of target is 6-28 in one-color and superset conditions (a; d), 6-18 in two-color condition (b), and 6-14 in three-color condition (c). To make graphs more readable, original data were pooled every two target numbers. (e-h) Scatter and linear regression results in Experiment 1C.

**2.2 Experiment 2**

The mean of estimation is not significantly different, either between the baseline and the intermingled conditions, *t*(11) = 1.79, *p* = .100, *d* = 0.52, *BF_10_* = 0.99; or between the baseline and the blocked conditions, *t*(11) = 1.19, *p* = .258, *d* = 0.34, *BF_10_* = 0.52.

In the intermingled condition there is no significant difference among 3 sessions, *F*(2, 22) = 0.875, *p =* .431, *η^2^* = 0.07, BF_10_ = 0.37. In the blocked condition, there is a significant difference among sessions, *F*(2, 22) = 3.677, *p =* .042, *η^2^* = 0.25, BF_10_ = 2.29. Difference exists between Session 2 and 3, *t*(11) = 2.70, *p* = .021, *d* = 0.78, *BF_10_* = 3.31, and between Session 1 and 3, *t*(11) = 2.16, *p* = .054, *d* = 0.62, *BF_10_* = 1.57.
